# A review on surface functionalization of carbon nanotubes: methods and applications

**DOI:** 10.1186/s11671-023-03789-6

**Published:** 2023-02-13

**Authors:** Eid M. Alosime

**Affiliations:** King Abdulaziz City for Science and Technology, P.O. Box 6086, Riyadh, 11442 Saudi Arabia

**Keywords:** Carbon nanotubes, Composites, Radiolytic functionalization, Catalyst, *γ*-irradiation, Fuel cell

## Abstract

In this *review*, the radiolytic and physical methods that can be used for the functionalization of carbon nanotubes (CNTs) and their applications as a support for fuel cell electrodes are described. Alloy nanoparticles have also been examined. For example, Pt–Ru nanoparticles were deposited onto a functionalized multiwalled carbon nanotube (MWNT) composite by reducing metal ions (e.g., Pt4+ and Ru3+) here using γ-irradiation and, hence, creating Pt–Ru/MWNT catalysts. The morphology, size, and composition of these Pt–Ru/MWNT catalysts were characterized by transmission electron microscopy (TEM), X-ray diffraction (XRD), and elemental analysis, respectively. The efficiency of the Pt–Ru/MWNT catalyst was examined for use in the oxidation of carbon monoxide (CO) and methanol. The results of stripping voltammetry for the adsorbed CO on the Pt–Ru/MWNT catalyst electrodes indicated that CO oxidation was energetically favorable at these electrodes. Thus, Pt–Ru/MWNT catalysts were found to be suitable for electrode assembly in direct methanol fuel cells.

## Introduction

Since their discovery in 1991 [1], carbon nanotubes (CNTs) have attracted immense interest because of their unique mechanical and electric properties. CNTs have been applied in field emission devices [2], nanoelectronic devices [3], probe tips [4], reinforced materials [5, 6], hydrogen storage [7], and in other applications [8]. In several of these applications, it is crucial to have reactive functional groups on single-walled CNT (SWNT) and multiwalled CNT (MWNT) surfaces. However, CNTs are not actually applied in industries because of the bundle effect and van der Waals force induced on individual CNTs. As a result, CNTs form a bundle during their synthesis and purification. CNTs with a hexagonal graphite structure exhibit a large surface area and sliding effect. The thermal properties of CNTs are affected by tuning their composition using polymers or metals. Hence, the functionalization of CNTs was investigated in light of this property.

Attaching functional groups or aliphatic carbon chains to the outer surface of CNTs can dramatically increase the solubility and controlling ability of nanotube materials. CNT functionalization was mainly conducted using a chemical method [9–11]. Few studies have reported the functionalization of CNTs using *γ*-irradiation.

**Fig. 1:**
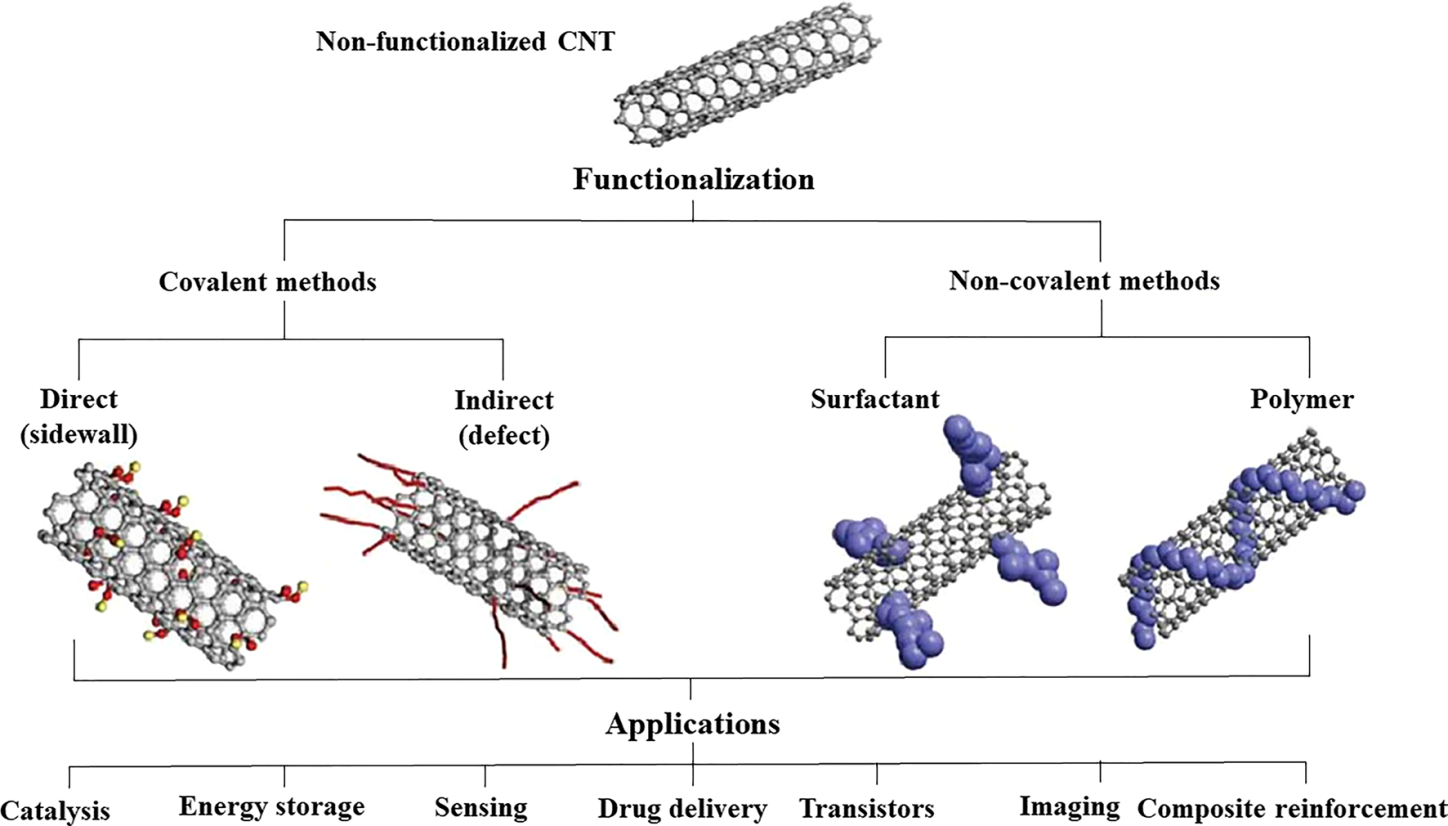
Functionalization methods and applications of carbon nanotubes

Generally, CNT surface modifications can be achieved through covalent or noncovalent functionalization methods. Figure 1 shows some of the common functionalization methods and applications of CNT. Different routes, including noncovalent and covalent modification of the outer surface, the filling of CNT channels, and the substitution of atoms, can all help to modify CNTs. Previous studies on modifying CNTs’ chemical structures to separate and stabilize them in a medium were conducted using direct sidewall functionalization [9]. One of the major benefits of the covalent interactions of CNTs is that they provide stronger bonds than noncovalent functionalization. However, the damage to sidewalls during covalent functionalization alters CNTs’ electronic structure, leading to the irreversible loss of double bonds [5–10]. Moreover, CNTs are not ideal nanostructures because 3% of defects can occur during synthesis. Defects can also be introduced into nanotubes during the purification and preparation stages. The oxygenated sites on nanotubes are also considered defects that can be functionalized through chemical oxidation through the use of gaseous oxidants or wet chemistry.

By using wet chemical processes, Tsang et al. [12] proposed a general approach for opening CNTs at the end and filling them with a variety of metal oxides. The procedure sparked extensive study into the physics and chemistry of loaded nanotubes that might be useful in isolation, storage, catalysis, and the development of novel electrical and magnetic components. The difference in thermal etching speeds between nanoparticles and nanotubes allowed Ebbesen et al. [13] to thermally oxidize a raw cathode deposit created with helium gas to produce pure MWNTs. The output was, however, less than 1%. However, Li [14] has recently developed a technique for modifying CNTs on a nanoscale utilizing controlled electron beam irradiation.

Wide-open porous materials with high surface area and low porosity carbon materials can be used to boost the frequency responsiveness of electrochemical double-layer capacitors (EDLCs). Electrolyte ions can move swiftly through the electrodes as a result of their open form [15, 16]. It is known that several studies [16–18] have been published on high-frequency EDLCs based on vertically aligned CNTs or graphene nanosheets over conducting surfaces, including electrodes. The search for alternative methods to produce carbon-facilitated metal nanoclusters has received a lot of attention. In order to determine the electrochemical activity and cell efficiency of Pt-based catalysts in direct-methanol fuel cells (DMFCs), it is crucial to consider the particle size and granular saturation amount [19].

In the present review, the functionalization of CNTs by noncovalent bonding and radiolytic irradiation is described. In addition, the radiolytic deposition of a metallic catalyst on functionalized CNTs as a support for use in a DMFC electrode is described.

## Functionalization of CNTs by noncovalent bonding

Functionalization by noncovalent bonding can occur through hydrogen bonding, van der Waals forces, charge transfer, dipole–dipole moment, stacking interactions, and so forth. This method is advantageous because functionalization occurs without a change in the carbon structure; therefore, the physical properties of CNTs (e.g., optical or electronic properties, etc.) are preserved, and this is important for biomedical applications of CNTs in imaging and other applications. Surfactants, hydrocarbons, aromatic hydrocarbons, biomolecules, and so forth are used for the functionalization of CNTs via noncovalent bonds; hence, CNTs are stabilized in an aqueous solution.

### Functionalization of CNTs using aromatic hydrocarbons

**Fig. 2:**
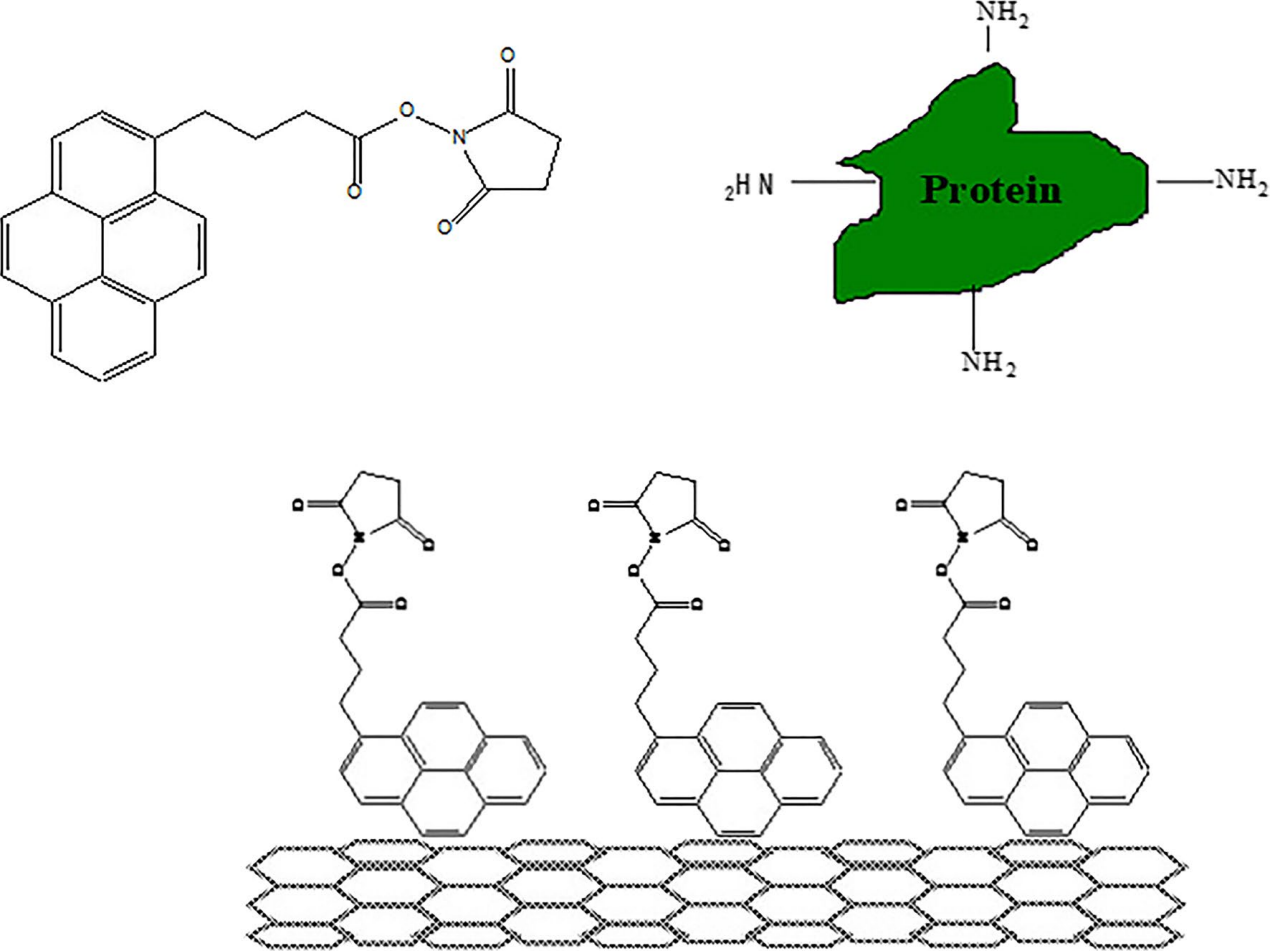
1-Pyrenebutanoic acid, succinimidyl ester 1 irreversibly absorbing onto the sidewall of a SWNT [12]

Among the most frequently used methods for CNT functionalization, one method involves the use of aromatic hydrocarbons. The CNT surface structure is composed of six-angle graphite; thus, pyrene, porphyrin, and a conjugated polymer interact with the CNT surface via stacking interactions. Chen et al. [20] were the first to report the immobilization of proteins on a CNT surface using pyrene as the anchoring agent (Fig. 2).

Pyrene is an aromatic hydrocarbon with four fused benzene rings, and its structure is similar to that of graphite, which has a lamellar structure. Chen et al. [20] adsorbed pyrene on a CNT surface with hydrophobic properties in dimethylformamide (DMF) and methanol as the organic solvents, followed by the adsorption of ferritin and Au nanoparticles on pyrene-immobilized CNTs. Hence, the materials were stabilized on the functionalized CNT surface via noncovalent bonds. Inorganic nanoparticles, small molecules with desirable properties, and polymerizable substances are only a few of the diverse species to which this straightforward process can be applied. The method could lead to stable SWNT concentrations in solutions, enabling the self-assembly of nanotubes with unchanged sp^2^ geometries and electrical properties. Petrov et al. [21] also reported the functionalization of CNTs by the noncovalent bonding of methyl methacrylate (MMA) with pyrene. Pyrene-modified MMA was made to interact with the CNT surface, resulting in the enhanced dispersion properties of CNTs because of the interaction of the MMA with the solvent. This method was employed to disperse the CNTs into an aqueous solvent and an organic solvent. Styrene, acrylic acid (AAc), and so forth were the monomers used in the abovementioned method [22, 23]. However, the surface modification of MWNTs using pyrene-containing polymers of different nature and molecular architecture is a very straightforward and efficient method for making them dispersible in water, in a variety of organic solvents, and for preparing homogeneous CNT-based polymer composites [21].

Several studies have reported the use of porphyrin for the functionalization of CNT surfaces via nonbonding covalent bonds [23–25]. Porphyrin can be complexed with Fe, Mg, Zn, and so forth. Porphyrin with an aromatic ring structure can also be adsorbed on CNT surface π–π interactions. Hence, porphyrin complexed with metal can be adsorbed on the CNT surface via noncovalent bonding. Furthermore, the modified porphyrin (conjugated porphyrin) can produce characteristic properties, rendering the dispersion of functionalized CNTs in various solvents. Water soluble porphyrin/CNTs can be aligned on poly(dimethylsiloxane) stamps and transfer printed onto silicon substrates, which may be useful for future device fabrication [24].

Meanwhile, conjugated polymers, such as poly(metaphenylene vinylene), PmPV, poly(arylene-thynylene)s, and poly[(m-phenyleneethynylene)-alt-(p-phenyleneethynylene)] (PPE) with a benzene ring in the polymer backbone can be functionalized by wrapping the conjugated polymer onto a CNT surface [26–28]. Star et al. [26] synthesized PmPV and measured the conductivity of a pure PmPV–CNT composite. The conductivity of the CNT composite was eight times greater than that of pure PmPV. When utilizing UV/Vis spectroscopy, the authors recognized that the region of the high conductivity for CNT–PmPV composite was reduced from π–π interaction on the CNT surface. They also measured the photovoltaic properties of the PmPV–CNT composite, revealing enhanced photon effects because of the perfect interaction of PmPV and CNT. This CNT composite may be used for molecular switching and molecular actuators. J. Chen et al. [28] also reported that the distance from the CNT surface to the PPE is controlled because of the rigidity of the PPE chain; hence, the solubility of CNTs is controlled by the π–π interaction distance. Star et al. [27] found that the process leads to an enhancement in solubility more than 20 times that of small-diameter SWNTs, hence allowing for the superior control of the relative placement of functionalities on the nanotube surface.

### Functionalization of CNTs by hydrophobic/hydrophilic properties

The functionalization of CNTs using surfactants and block polymers can be exploited via hydrophobic interactions and van der Waals interactions. As an extremely simple method, surfactants have been used for CNT functionalization for a long time. The hydrophilic head of the surfactant faces the solvents, while the hydrophobic tail of the surfactant faces the CNT surface [29, 30]. CNTs with hydrophobic properties have minimum contact with the aqueous solvent; therefore, this aqueous solution method is efficient for CNT functionalization. Cationic, anionic, and nonionic surfactants, such as sodium dodecyl sulfate (SDS), Tween 20, Triton X-100, sodium dodecylbenzenesulfonate, and octadecyl-trimethylammonium bromide, have been used [31–34]. Islam et al. [33] compared dispersion properties between CNTs modified with an anionic surfactant and CNTs modified with a nonionic surfactant according to the adsorption patterns and head and tail properties of the surfactant. The results from these studies have revealed that the dispersion of the CNTs was enhanced by using a long alkyl main chain and benzene ring. They also reported that to be making the bundle of CNT is protected using surfactant with small head and ionic characteristic properties because it creates the high packing density and charge–charge repulsion power.

Richard et al. [34] also confirmed the self-assembly of SDS on a CNT surface using transmission electron microscopy (TEM) imaging. Arrays of anionic and cationic surfactants, such as SDS, are formed by wrapping the surfactant on the CNT helical structure, whereas nonionic surfactants are coated only on the CNT surface via *π*–*π* interactions without any array patterns. Hence, nonionic surfactants only decrease the surface energy.

Moreover, block polymers with hydrophobic and hydrophilic sites can be used for CNT functionalization, including polystyrene-block-poly(ethylene oxide) (PS-PEO), polystyrene-block-poly(4-vinylpyridine) (PS-*b*-P4VP), and polystyrene-block-poly(acrylic acid) (PS-*b*-PAA) [33, 35, 36]. Similar to surfactants, block copolymers enhance the dispersion properties of CNTs because the hydrophobic site interacts with the CNT surface, such as van der Waals forces, while the hydrophilic site faces the solvent. Kang et al. [36] prepared CNTs as the core and a PAA ball as the shell using a PS–PAA block copolymer in DMF as the solvent without water. This method was advantageous because it could disperse CNTs in water. However, micelles made from PS-*b*-PAA were found to be able to improve the compositing of CNTs in a wide variety of polymer materials for structural, electronic, and thermal applications [36].

### Functionalization of CNTs using biopolymers

The functionalization of CNTs using biopolymers, such as DNA, RNA, peptides, enzymes, and so forth, has been achieved via hydrogen bonding, van der Waals forces, charge transfer, dipole–dipole moments, π–π stacking interactions, and so forth. The functionalization of the CNT surface using biopolymers can be conducted only in an aqueous solution because of the characteristic properties of biopolymers, and the resultant functionalized CNTs can be used in biosensing, drug delivery, and so forth. Nucleic acid is one of the most interesting biomolecules because of its widespread applications, including nanoscale devices, gene therapy, nucleic acid sensing, and the easy structure control effect. Sometimes, large-sized DNA is wrapped on the CNT surface as a helical structure, whereas small-sized DNA is inserted into a CNT hole. Zheng et al. [37] reported that single-strand DNA is wrapped on the CNT surface in a helical form; hence, the helical structure changes according to the CNT diameter and chirality of the DNA; they also employed ion exchange chromatography to separate metallic CNTs and semiconductor CNTs because of their electric properties [38, 39]. As reported previously, RNA is more interesting and advantageous than DNA because of no mutation, easy transfer of the respective material in mammals because of the absence of opportunistic recognition, and easy removal using ribonuclease (RNase) as the enzyme. Jeynes et al. [40] reported the wrapping of total cellular RNA (tcRNA) on the CNT surface, finding dramatically enhanced dispersion compared with that in the case of SDS. The interaction concept was *π*–*π* stacking interaction and van der Waals force. Jeynes et al. [40] also revealed that CNT purification is possible via the compact removal of wrapped RNA using Rnase.

**Fig. 3:**
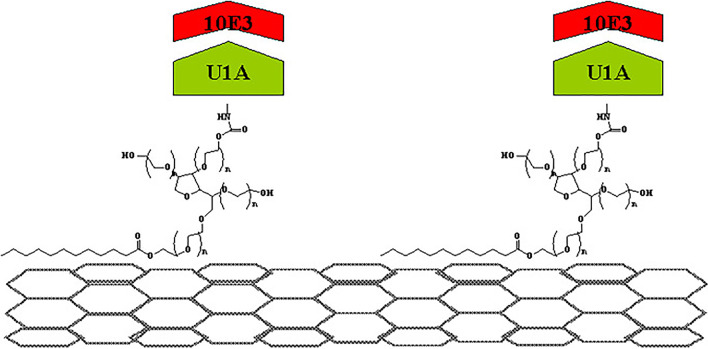
Specific detection of mAbs binding to a recombinant human autoantigen [41]

Among biopolymers, large-sized proteins can be used for CNT functionalization. Proteins with a complex structure and various functional groups can render various and complex interactions to CNTs, such as electron interactions, hydrophobic interactions, van der Waals interactions, cationic combinations for amine groups, and hydrogen bonding. The hydrophilic/ion characteristic site is forced to the aqueous solution, whereas the hydrophobic/aromatic hydrocarbon site is forced to the CNT surface. Chen et al. [41] fabricated an electric biosensor for a specific probe protein in solution (Fig. 3). This is a crucial discovery because it allows for the applications of CNTs as sensors in various biofields, such as proteomics and pathognomy, for electric biodevices.

Meanwhile, peptides composed of the two amino acids can be used for CNT functionalization. CNTs can also control the sequence of peptides as DNA by using the amino acid. Peptides with a benzene ring, such as tryptophan, phenylalanine, and tyrosine, can interact with the CNT surface via electron interactions, and peptides with hydrophobic and hydrophilic properties can interact with the CNT surface via hydrophobic interactions such as surfactants [42, 43]. Dieckman et al. [44] also synthesized peptides through modeling and coated the synthesized peptide with CNTs. According to the above method, the CNT-wrapped peptide acted as a biosensor, and the array the CNT on the pattern antibody, which is the respective antibody. Therefore, the presence of the functionality substantially can affect the symmetric radial vibrations of the hollow cylinders. This concomitant mode stiffening can be attributed to the interactions between the CNTs and wrapped lipid molecules.

## Radiolytic functionalization of CNTs

Functionalization can be conducted using ionization radiation [45], UV [46], plasma [47], or chemical initiators [48]; among these methods, radiation-induced functionalization is one of the most effective techniques because of its uniform creation of radical sites in the respective matrix. Generally, two radiation functionalization techniques can be used: (1) the direct method, in which a respective substrate is immersed or dissolved in either an individual monomer with functional groups or a solution of monomers, which is followed by irradiation of the entire mixture, and (2) the preirradiation method, in which a substrate is activated by irradiation, regardless of the presence of oxygen, and sequentially reacted with the monomer.

### Functionalization of CNTs by *γ*-irradiation

The functionalization method of CNTs using *γ*-irradiation was first reported in the literature [49], where X-ray irradiation was used to modify CNTs and where SWNTs and MWNTs were subsequently functionalized in aqueous and nonaqueous media. Peaks observed at ~ 1700 and 3400 cm^−1^ in the FTIR spectra of the *γ*-irradiated SWNTs and MWNTs corresponded to carboxylic acid and hydroxyl groups, respectively. Furthermore, sulfonic acid groups were introduced into the MWNTs and SWNTs by using thiosulfite or carbon disulfide as the sulfur source, which was confirmed by the presence of a characteristic peak at ~ 1124 cm^−1^ in the FTIR spectrum of the modified MWNTs or SWNTs. In addition, alkyl groups could be introduced into the MWNTs or SWNTs using Triton X-100 as the dispersing agent in an organic solvent. Plausible mechanisms for the generation of functional groups on the nanotube surface were proposed [49].

Glycidyl methacrylate (GMA) is a monomer that is easily modified into various functional groups. With the polymerization of GMA, the epoxy groups in poly-GMA can be modified to alcohol [50], amine [51], phosphonic acid, sulfonic acid [52], and so forth. [53, 54]. The epoxy group of poly-GMA can be generated onto a CNT surface by radiation-induced polymerization. MWNTs with various amine groups were prepared by the radiation-induced polymerization of GMA onto MWNT and the subsequent amination of poly-GMA graft chains. The physical and chemical properties of the GMA-grafted MWNT and aminated MWNT have been investigated using IR, SEM, XPS, and TGA. However, Chung et al. [54] reported that the increased diameter of the MWNTs indicated the successful attachment of epoxy groups by the radiation-induced graft polymerization of GMA. Amines can be covalently introduced onto MWNT supports with epoxy groups through an SN_2_ reaction. They concluded that the aminated MWNT supports could be used in enzyme-immobilized biosensors as good electron transfer materials and as supports for enzyme immobilization.

**Fig. 4:**
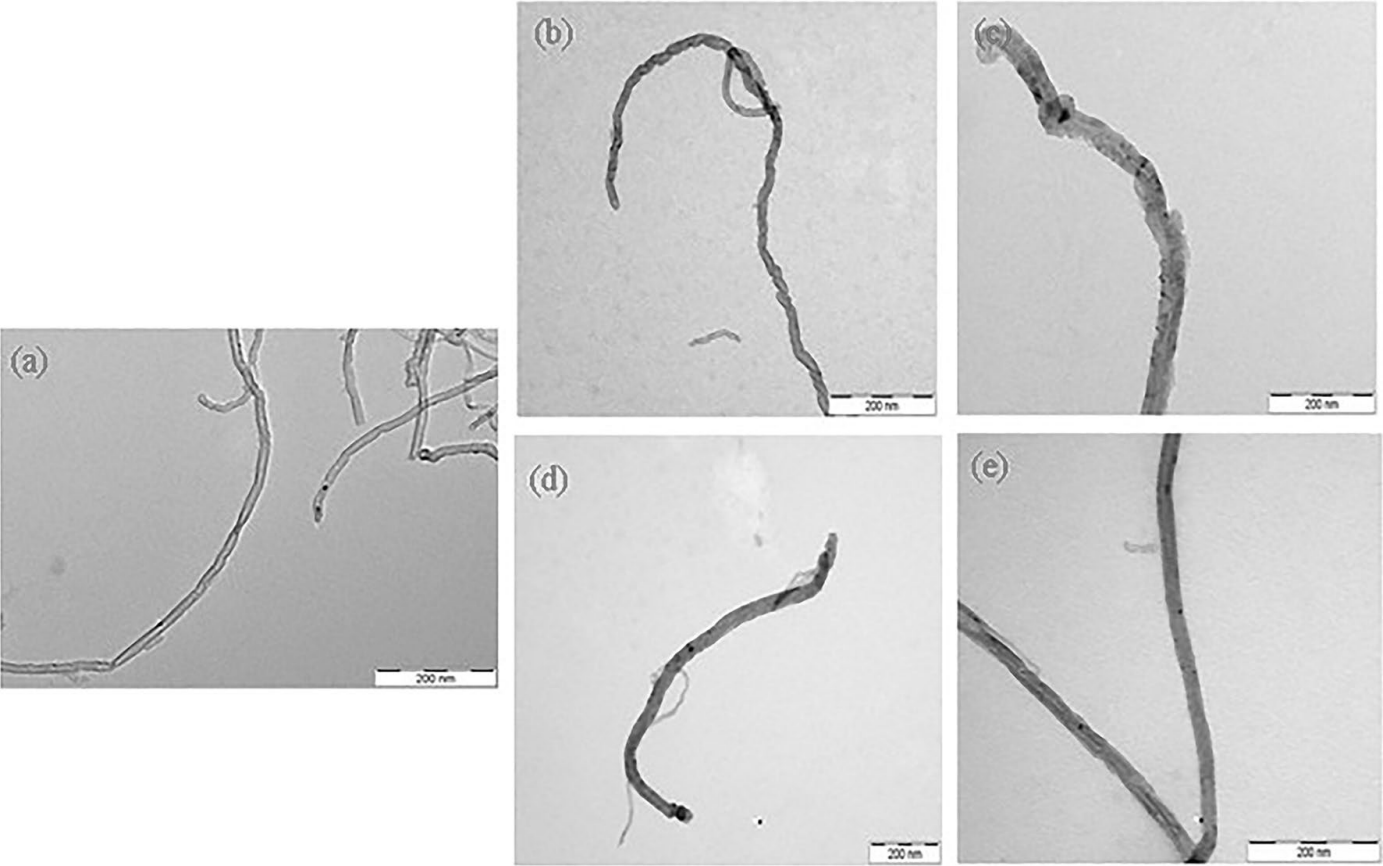
TEM images of the purified MWNT (**a**), AAc-grafted MWNT (**b**), Man-grafted MWNT (**c**), GMA-grafted MWNT (**d**), and Mac-grafted MWNT prepared by γ-irradiation [5]

Several studies have reported radiation-induced polymerization using a polymer matrix to modify the hydrophobic properties of the polymer matrix to hydrophilic properties [55–57]. Radiolytic polymerization exhibits several advantages compared with conventional chemical/photochemical polymerization, as follows: (1) Reaction rates that can be controlled as radiation have been well defined. (2) Radiation can be employed with any solvent; furthermore, the reaction can be conducted without a solvent. (3) Radicals, as the reaction site, can be uniformly generated on the substrate. (4) Radiation-induced polymerization can be performed at an ambient temperature. (5) The radiation reaction occurs in one pot, and byproducts are not produced. Nevertheless, few studies have investigated the radiolytic functionalization of CNTs using monomers with functional groups [54, 55]. A MWNT was functionalized by the radiation-induced polymerization of various vinyl monomers, such as AAc, GMA, methacrylic acid (MAc), and maleic acid (MA), using *γ*-irradiation in an aqueous solution as a one-step reaction (Fig. 4).

### Functionalization of CNTs by the radiolytic deposition of metallic nanoparticles and their applications for DMFC

**Fig. 5:**
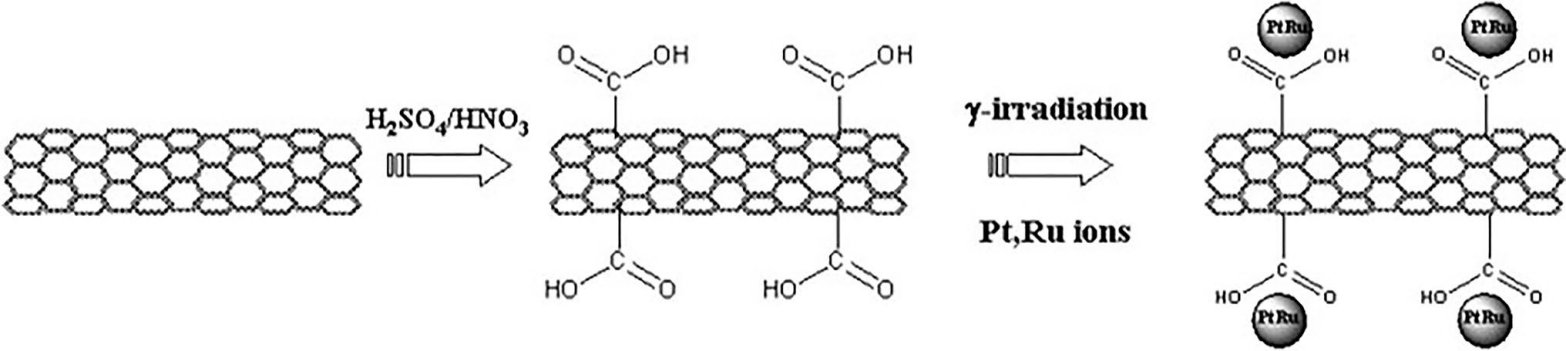
Surface modification of SWNTs through the presence of characteristic peaks of carboxyl and hydroxyl groups and providing support for the dispersion of Pt-Ru on the surface of SWNTs

CNTs can be functionalized by the radiolytic deposition of metallic nanoparticles using γ-irradiation. The nanoparticles of metals (e.g., Ag, Pd) and an alloy (Pt–Ru) were dispersed on SWNTs as the support materials using γ-irradiation at room temperature. Notably, the attachment of the nanoparticles onto SWNTs was sufficiently strong, even after chemical cleaning and ultrasonication. FTIR spectroscopy provided evidence not only for the surface modification of SWNTs through the presence of characteristic peaks of the carboxyl and hydroxyl groups, but also for support in terms of the dispersion of nanoparticles on the SWNT surface (Fig. 5) [58].

Pt–Ru alloy particles were successfully dispersed onto various carbon structures, including Vulcan XC-71, Ketjen-300, Ketjen-600, SWNTs, and MWNTs, to form new Pt–Ru/carbon-based composites. Pt–Ru alloy nanoparticles were mainly aggregated on Vulcan XC-71, Ketjen-300, and Ketjen-600, while SWNTs and MWNTs provided hydrophilic sites for improving the distribution of alloy particles. FTIR spectroscopy confirmed the formation of carboxylate and hydroxyl groups on the nanotube surface after *γ*-irradiation. These functional groups helped serve as nuclei rendering the improved dispersion of alloy nanoparticles onto SWNTs and MWNTs [59]. However, metallic alloy nanoparticles aggregated on the surfaces of the carbon supports because of their hydrophobic nature, which was overcome by modifying the surface of the carbon support to give it hydrophilic properties.

**Fig. 6:**
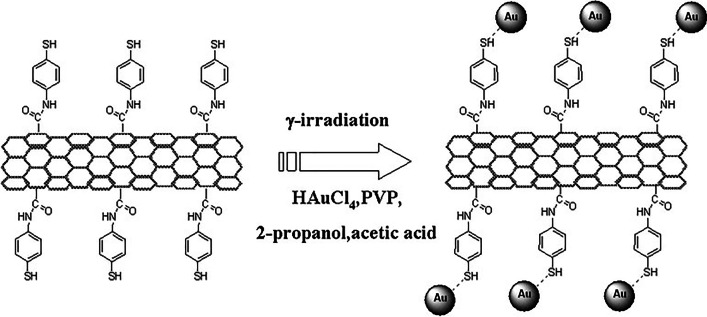
Preparation procedure for Au@MWNT catalyst by γ-irradiation for fuel cell

On the other hand, Au nanoparticles were dispersed into thiol-functionalized MWNTs using *γ*-irradiation (Fig. 6) [60]. The thiol groups were used as a linker to hold the gold nanoparticles without agglomeration. Field emission TEM, UV–visible spectroscopy, and XRD analysis were employed to confirm the existence of Au metallic particles in the MWNT matrix. *γ*-irradiation was used as a source to reduce gold metal ions without having any additional reducing agents. The method can provide the formation of gold nanoparticles without being contaminated by the byproducts from the normal reducing agents [60].

**Fig. 7:**
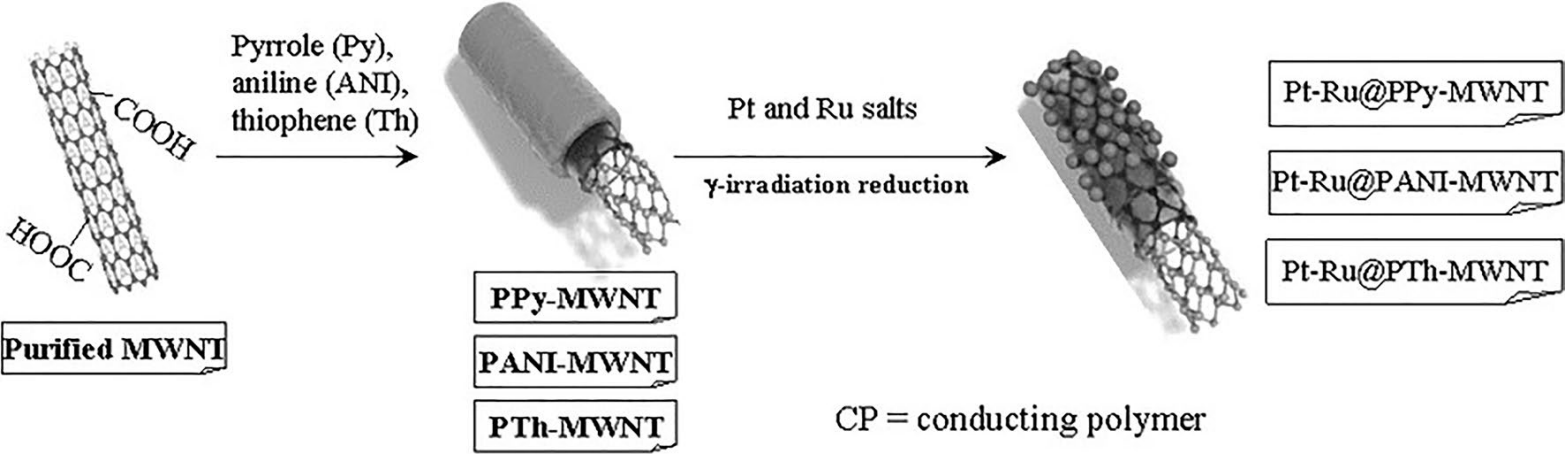
Preparation procedure for Pt-Ru@MWNT catalyst by various reduction method for fuel cell

However, a large number of metallic catalysts on the CNT surface cannot be deposited using γ-irradiation because the CNT surface exhibits hydrophobic properties. Therefore, a polymer with hydrophilic/hydrophobic properties was wrapped on the backbone on the CNT surface using in situ polymerization. Subsequently, metallic catalysts on polymer-functionalized CNTs using *γ*-irradiation were deposited as follows: (1) Three polymers, that is, poly(*ε*-caprolactone), PCL, poly(maleic anhydride), PMA, and poly(pyrrole), PPy, were coated on the MWNT surface by in situ polymerization, respectively. Pt–Ru nanoparticles were deposited on the polymer–MWNT composite by *γ*-irradiation reduction (Pt–Ru@polymer–MWNT catalyst). The as-prepared Pt–Ru@polymer–MWNT catalysts were successfully confirmed by TEM, XRD, and elemental analysis. The efficiency of the Pt–Ru@polymer-MWNT catalyst was examined for CO stripping and methanol oxidation. The Pt–Ru@PPy-MWNT catalyst exhibited a high adsorption capacity for CO and high catalytic efficiency for methanol oxidation. Hence, the Pt-Ru@polymer-MWNT catalyst can be used as a DMFC electrode [61]. (2) To prepare Pt–Ru@PPy–MWNT electrocatalysts with high efficiency, the Pt–Ru@PPy–MWNT catalysts were prepared using different approaches. As anchoring materials, PPy was coated on the surface of the MWNT using in situ polymerization of Py in the presence of a MWNT (PPy–MWNT composite). Subsequently, Pt–Ru nanoparticles were deposited onto the PPy–MWNT composite by reducing the metal ions through γ-irradiation without a reducing agent (Pt–Ru@PPy catalyst) to prepare an electric catalyst for DMFC. Pt–Ru nanoparticles were also decorated on the Ppy–MWNT composite by chemical reduction using formaldehyde as the reducing agent with stirring using a magnetic bar, which was done with the assistance of microwave irradiation and ultrasonic irradiation. The size, morphology, and composition of Pt–Ru@PPy–MWNT catalysts were determined by TEM, XRD, and elemental analysis. The size of the Pt–Ru nanoparticles on the PPy–MWNT composite prepared by *γ*-irradiation were greater than that of the Pt–Ru nanoparticle prepared by chemical reduction. The Pt contents on the Pt–Ru@PPy–MWNT catalysts prepared by *γ*-irradiation and chemical reduction with stirring and ultrasonication were greater than the Ru contents, while the Ru contents were greater than the Pt contents for the Pt–Ru@PPy–MWNT catalyst prepared using chemical reduction assisted with microwave irradiation. The efficiency of the Pt–Ru@PPy–MWNT catalyst was examined for CO stripping. The stripping voltammograms for the adsorbed CO on the Pt–Ru@PPy–MWNT catalyst electrodes prepared by γ-irradiation revealed that CO oxidation was energetically favorable at these electrodes. Thus, the Pt–Ru@PPy–MWNT catalysts prepared by γ-irradiation were found to be suitable for electrode assembly in DMFCs [62]. (3) The Pt–Ru@CP–MWNT catalysts were prepared by the radiolytic deposition of Pt–Ru nanoparticles on a conduction polymer (CP)-coated surface of the MWNT. Three conducting polymers, that is, PPy, polyaniline (PANI), and polythiophene (PTh), were coated on the surface of the MWNT using in situ polymerization of the respective monomers in the presence of MWNT. The Pt–Ru nanoparticles were deposited onto the CP–MWNT composite by a reduction of metal ions using γ-irradiation, affording the Pt–Ru@CP–MWNT catalysts. SEM, TEM, and elemental analysis were employed to analyze the size, morphology, and composition of the Pt–Ru@CP–MWNT catalysts. The diameter of the CP–MWNT composite was 10–70 nm greater than that of the crude MWNT. TEM images of the Pt–Ru@CP–MWNT catalyst provided clear evidence for the dispersion of the Pt–Ru alloy nanoparticles on the CP–MWNT composite surface compared with the crude MWNT surface, which can be attributed to the change from the hydrophobic surface of crude MWNT to a more hydrophilic polymer-coated MWNT catalyst surface. The efficiency of the Pt–Ru@CP–MWNT catalyst was examined for CO stripping and methanol oxidation. Compared with the Pt–Ru@PTh–MWNT catalyst, the Pt–Ru@PPy–MWNT catalyst and Pt–Ru@PANI–MWNT catalyst exhibited enhanced activities for the electrooxidation of methanol. In addition, stripping voltammograms for the adsorbed CO on the Pt–Ru@CP–MWNT catalyst electrodes revealed that CO oxidation was energetically favorable at these electrodes. Thus, the Pt–Ru@CP–MWNT catalysts were found to be suitable for electrode assembly in DMFCs (Fig. 7) [63].

## Conclusions

The functionalization of CNTs by physical and radiolytic methods has been described. Furthermore, the radiolytic deposition of Pt–Ru nanoparticles on functionalized CNTs as a support for use in DMFC electrodes was discussed.

The functionalization of CNTs via noncovalent bonding can be advantageous because the CNT structure is not damaged. In addition, various functional groups can be introduced to the as-functionalized CNTs. However, some issues regarding solvents to use the composite, electric devices, and biofield still exist. Recently, CNTs have been extensively investigated in biosensing because of their facile functionalization, which can be easily conducted by noncovalent bonding compared with using a synthetic polymer, and the CNTs demonstrated wide application potential as biosensors. The application of functionalized CNTs is also expected in biosensing because functionalized CNTs are cost-effective and because CNTs with specified biomolecules such as DNA, protein, and antigen/antibody as making devices probe the molecules.

On the other hand, various functional groups can be introduced to the CNT surface using a radiation technique in a one-step process. This method is extremely simple for preparing polymer–CNT composites, which can be used as supports for the deposition of metallic catalysts by *γ*-radiation in fuel cells. This radiolytic functionalization of CNTs is expected to be an actual industrial application because the method is extremely simple, cost-effective, and continuous and is conducted at room temperature, with or without solvents.

## Data Availability

Not applicable.
